# Field Epidemiology, 2nd edition

**DOI:** 10.3201/eid0902.020697

**Published:** 2003-02

**Authors:** Marc A. Strassburg

**Affiliations:** University of California and Los Angeles County Department of Health Services, Los Angeles, CA

Before the first edition of Field Epidemiology was published in 1996, no single authoritative textbook or manual existed for public health epidemiologists engaged in active field investigation and outbreak control. Those needing preparation or guidance in responding to acute problems in the community (infectious or noninfectious) were left to read a variety of disparate materials and exercises (many from the Centers for Disease Control and Prevention [CDC]), learn on the job, or find a mentor.

In the current revised and augmented edition of Field Epidemiology, the first chapter defines field epidemiology as “the constellation of problems faced by epidemiologists who are called upon to investigate urgent public health problems…” and further states, “public health epidemiologists must travel to and work in the field to solve the problem.” Thus, for those practicing in this arena, the motivation is not primarily research oriented but rather geared to those problems for which government agencies usually are given the primary mandates and responsibilities. I would imagine that those involved in “nonacute” epidemiology or investigating non-urgent public health problems also consider themselves to be carrying out “field work” (such as doing a nutritional or behavioral survey or assessment in the community) and might object to the all-inclusive title used for this book. Perhaps the title of “The Field of Acute Public Health Epidemiology,” although awkward, is a better description of the content of this book.

How will readers of Emerging Infectious Diseases benefit from such a book? Clearly this book provides an excellent primer in basic epidemiologic techniques, including discussions of principles and surveillance; collecting, analyzing, interpreting, and presenting data; surveys and sampling; even the more advanced topics, such as confounding, the role of chance, and the exploration of interactions, are discussed. More importantly, I believe the many chapters describing the operational aspects of epidemiologic field investigations—including the legal considerations, special problems in health-care settings, current public health laws, and laboratory support—provide insights into both the methods and investigational steps for many of the diseases that eventually result in publication in journals like Emerging Infectious Diseases. Thus, such a book provides readers with a greater appreciation and understanding for work behind the resulting publication.

Overall, I find the writing to be lucid and readable. Moreover, one can read any chapter independently. A large number of examples and case studies are used. For example, before the reader has progressed more than a sentence or two into the book, the authors describe the events that unraveled at the American Legion Convention in Philadelphia, which led to the discovery of *Legionella pneumophila*. Throughout the book, chapters focus on practical and real-world situations.

Over 25 years ago, I started work in Los Angeles as an epidemiologist in the Acute Communicable Disease Control program and later for the Toxics Epidemiology program. This book would have been worth its “weight in gold” if it had been available to me as a novice epidemiologist. Even today, 200–300 outbreaks and investigations later, I found much to learn, particularly those chapters providing new and updated information on bioterrorism preparedness and response and field investigations of occupational disease and injury. I especially found intriguing the chapter on surveillance, which includes a number of case studies, giving the reader an appreciation for the many biases and limitations in analyzing and interpreting incidence and prevalence data. The book includes an excellent appendix with current interesting Web sites. Despite the large number of authors, almost all experts in their fields (a veritable who’s who of well-respected and accomplished epidemiologists), with slightly different writing styles, most of the book flows evenly. Some oddities do appear now and then, such as the mnemonic or acronym called “SLACK OFF” used in the chapter on analysis. This term is meant to help the investigator remember to describe the methods and techniques in an investigation (S is for Shells, which actually means setting up tables for analysis at the start of an investigation). I found the chapter on sampling lacking in examples, unlike most chapters in the book. Particularly well-written and of interest to the computer and technologically minded is the chapter by Andy Dean, the father of Epi Info Software. This robust freeware product provided by CDC is one of the best-kept secrets of epidemiologists, and researchers in allied fields can benefit from reading about this comprehensive database and statistics software for public health professions (available from: URL: http://www.cdc.gov/epiinfo).

Overall, this comprehensive volume can be viewed as a prolegomenon for any future public health epidemiologist. I highly recommend it for those who will be practicing field epidemiology and for those who wish to know more about the application of epidemiologic methods to acute and urgent public health problems.

